# Association between blood urea nitrogen levels and diabetic retinopathy in diabetic adults in the United States (NHANES 2005-2018)

**DOI:** 10.3389/fendo.2024.1403456

**Published:** 2024-05-10

**Authors:** Kejie Du, Wenjuan Luo

**Affiliations:** Department of Ophthalmology, The Affiliated Hospital of Qingdao University, Qingdao, China

**Keywords:** diabetic retinopathy, blood urea nitrogen, diabetes, complication, NHANES

## Abstract

**Objective:**

To investigate the association between blood urea nitrogen (BUN) levels and diabetic retinopathy (DR) in adults with diabetes mellitus (DM).

**Methods:**

Seven cycles of cross-sectional population information acquired from NHANES(national health and nutrition examination surveys) 2005-2018 were collected, from which a sample of diabetic adults was screened and separated into two groups based on whether or not they had DR, followed by weighted multivariate regression analysis. This study collected a complete set of demographic, biological, and sociological risk factor indicators for DR. Demographic risk factors comprised age, gender, and ethnicity, while biological risk factors included blood count, blood pressure, BMI, waist circumference, and glycated hemoglobin. Sociological risk factors included education level, deprivation index, smoking status, and alcohol consumption.

**Results:**

The multiple regression model revealed a significant connection between BUN levels and DR [odds ratio =1.04, 95% confidence interval (1.03-1.05), *p*-value <0.0001],accounting for numerous variables. After equating BUN levels into four groups, multiple regression modeling showed the highest quartile (BUN>20 mg/dl) was 2.22 times more likely to develop DR than the lowest quartile [odds ratio =2.22, 95% confidence interval (1.69-2.93), *p*- value <0.0001]. Subgroup analyses revealed that gender, race, diabetes subtype, and duration of diabetes had a regulating effect on the relationship between BUN and DR.

**Conclusion:**

BUN levels were related with an increased prevalence of DR, particularly in individuals with BUN >20 mg/dl. These findings highlight the significance of BUN level in assessing the risk of DR.

## Introduction

Diabetic retinopathy (DR) is a frequent consequence of diabetes and a primary cause of visual loss in many populations (1). According to a recent nationwide survey in China (2), the morbidity of DR and sight-threatening DR (95% confidence intervals) was 16.3% (15.3%-17.2%) and 3.2% (2.9%-3.5%), respectively. The prevalence of DR varies according to geography, economic level, ethnicity, and so on (3), as well as the duration of diabetes (4), dietary habit (5), lifestyle (6), and blood pressure control (7). Although the prevalence is under control, the global population is, creating considerable public health challenges and economic burdens.

Blood urea nitrogen (BUN) measurement is an ancillary test that checks for glomerular filtration function and is frequently used to assess renal function. Numerous studies have demonstrated that the ratio of BUN to creatinine can be used to assess cardiac function (8) and that a higher BUN/creatinine ratio is correlated with a poor prognosis in individuals with chronic heart failure (9–12). The potential application of blood urea nitrogen has been continually investigated through further research. Higher BUN levels have been contacted with an increased risk of diabetes (13). Furthermore, BUN during pregnancy predicts the risk of DR (14), and BUN can be used independently to predict adverse cardiovascular events (15) and renal impairment following diabetes onset (16). While recent research have shown that BUN is associated with DR (16, 17). A strong association between BUN and DR has been concluded in several clinical studies in recent years on the risk of developing DR and risk factors for fibrovascular proliferation in DR. In the study by Shi Rong et al. (16), a DR risk line was designed and BUN was included to predict the risk of DR. The study by Wu et al. (17) identified BUN as an independent risk factor for fibrovascular proliferation in patients with proliferative diabetic retinopathy by univariate and multivariate analyses. Another study (18) showed that BUN is more closely associated to the risk of DR in persons with type 2 diabetes for shorter time. These studies have focused on the possible inextricable relationship between BUN and DR, but there is no literature focusing on the correlation between BUN and DR separately, and in-depth questions such as how BUN levels affect patients with different characteristics of DR have not been addressed. Our study designed to comprehensively evaluate the correlation between BUN level and DR, as well as whether a combination of BUN and other factors influences the likelihood of DR.

## Materials and methods

### Population included

**Figure 1:**
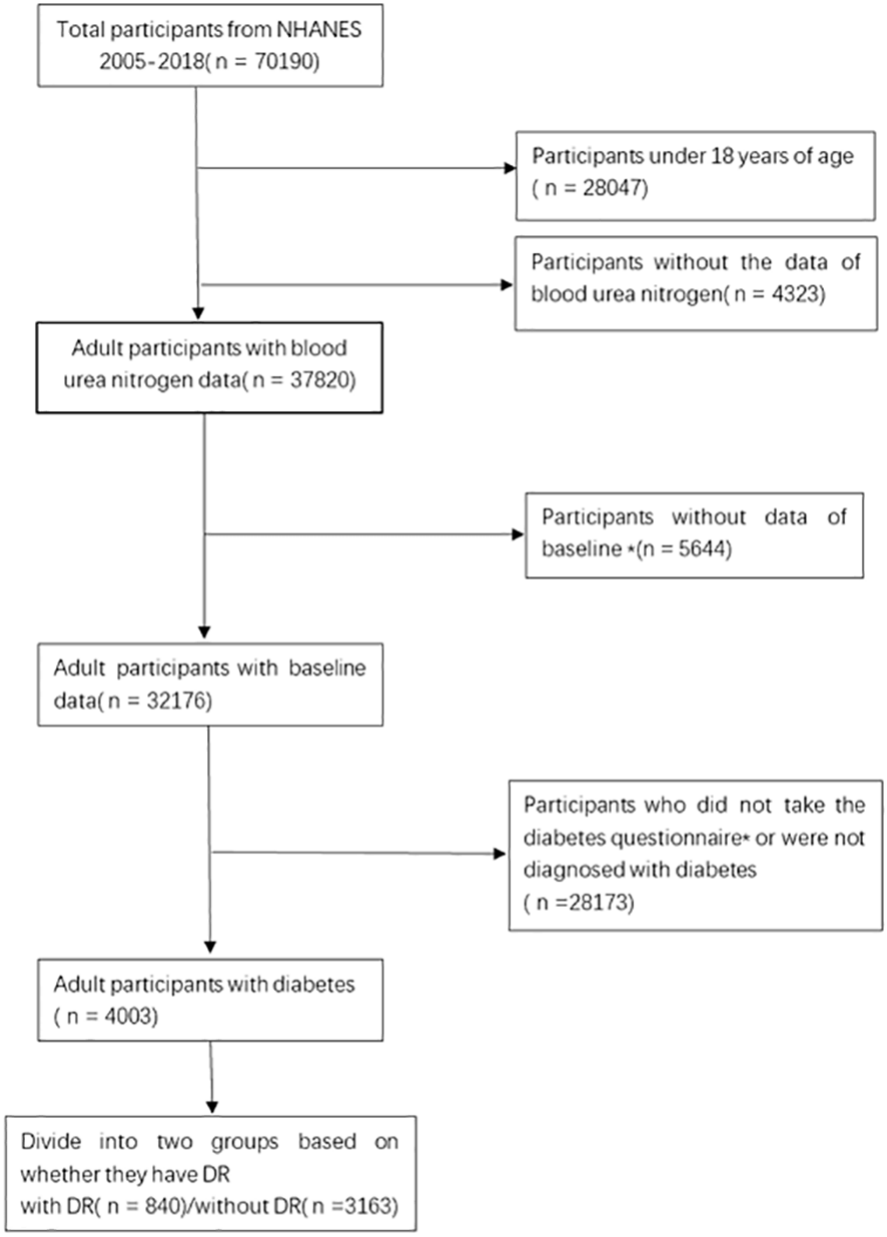
Flow chart of the selection process. *Baseline data includes age, gender, race, degree of education, poverty level, smoking status, drinking status, BMI. *Diabetes questionnaire includes whether they were diagnosed with diabetes and whether they had retinopathy, etc.

The information for this research were gathered from the NHANES database, which is a comprehensive and reliable database that collects extensive data on nationally representative research populations through multi-stage stratified samples. The NHANES official website presents the sampling, collection and statistical plan for all data. All data collection and statistics were approved by the Ethics Review Board(ERB), and the numbers or descriptions of the ethical review protocols for all survey cycles are available on the official website. The current study comprised 70,190 participants from the 2005-2018 data surveys, excluding 28,047 underage participants, 4,323 with missing BUN data, 5,644 without complete baseline data. We used the doctor’s diagnosis to determine whether the participant had diabetes. According to the questionnaire “{Other than during pregnancy, {have you/has SP}/{Have you/Has SP}} ever been told by a doctor or health professional that {you have/{he/she/SP} has} diabetes or sugar diabetes?”, 28,173 participants were not diagnosed as having diabetes, for a final population of 4003 participants in this survey (**Figure 1**).

### Determination of diabetic retinopathy

NHANES created a diabetes questionnaire with items that were administered by trained interviewers at home via a computer-assisted personal interview (CAPI) system. Some of the questions also used hand cards that indicated response categories. The handcards were read to the responder by the home interviewer if needed. Direct interviews were conducted with participants aged 16 and older, as well as liberated minors. Proxies provided information to survey participants under the age of 16 and those who were unable to answer the questions directly. The CAPI system was designed with internally installed conformance inspections to minimize data entry mistakes. CAPI also utilized an online help screen to aid interviewers in defining important terminologies from the questionnaire. To establish DR, Participants were given the diabetes questionnaire and were asked a question in the questionnaire “Has a doctor ever told {you/SP} that diabetes has affected {your/his/her} eyes or that {you/s/he} had retinopathy (ret-in-op-ath-ee)?”.

### Measurement of blood urea nitrogen levels

Individual serum samples were gathered for the determination of blood urea nitrogen levels during 2005-2018 NHANES surveys, and the steps of specimen collection, preservation, and processing were guided by laboratory/medical technicians throughout the process, with detailed laboratory method documentation provided in the NHANES database.

### Covariates

Based on the existing literature reports and medical expertise, we used statistical methods to evaluate the candidate variables and identify those variables that are related to the dependent variable as a way of ensuring the predictive value of these variables for the outcome. The demographic variables comprised in this research contained age, gender, race, poverty index, and educational level. Anthropometric variables comprised in the research included systolic blood pressure, diastolic blood pressure, BMI, waist circumference. And laboratory variables included glycosylated hemoglobin, serum albumin levels, triglycerides, total cholesterol, low-density lipoprotein (LDL) cholesterol, and high-density lipoprotein (HDL) cholesterol. Self-reported smoking and alcohol usage were also taken into account, as indicated by the questionnaires such as “whether you smoked at least 100 cigarettes”, “Whether you drank at least 12 glasses of wine per year” and “Age at diagnosis of diabetes”. “. Those who answered “yes” to the question “Do you drink at least 12 glasses of wine per year” were classified as drinkers. The NHANES website contains the computations for each of these covariates. In addition, the length of time with diabetes was computed using the age upon diagnosis of diabetes and the age at the time of the survey.

### Statistical analyses

We stratified the cohort of diabetic patients into two cohorts based on the presence or absence of diabetic retinopathy (DR). Descriptive statistics for normally distributed continuous variables were presented as mean ± standard deviation, and the statistical significance was assessed using the independent samples t-test. Categorical variables were expressed as proportions, and the χ2 test was utilized for assessing their significance.

The statistical analyses were performed using R (http://www.rproject.org). The overall sample was weighted by 2-year mobile testing center test weights (WTMEC2YR) acquiredfrom the NHANES database. Categorical variables by ratio, with *p*<0.05 indicating a statistically significant difference. Continuous variables were described by averages with standard deviations.

Blood urea nitrogen levels were divided evenly into four groups and with the lowest quartile serving as the reference quartile for determining the connection with DR. This study utilized a logistic regression model to estimate 95% confidence intervals (CI) and determine the relationship between BUN and DR using odds ratios (ORs). In addition, subgroup analyses were conducted by age at diagnosis of diabetes, gender (<=40/>40), ethnicity, and diabetes duration(quartiles) to examine how these risk factors affect the association. Model 1 didn’t modify any covariates, Model 2 corrected for age, gender, and race, while Model 3 adjusted for all covariates in this study.

## Results

### Baseline characteristics

This study comprised 4003 persons with diabetes mellitus with an average age of 61.46 ± 13.03 years.Of those, 52.24% were male and 47.76% were female. They were fallen into two groups based on the existence or absence of retinopathy, with the two groups having different demographic, sociological (**Table 1**) and biological features (**Table 2**). The study population description showed no statistical differences between the two groups in variables such as age, gender, race, smoking status, BMI, and waist circumference. Patients in the DR group had a higher BUN, serum albumin, creatinine, glycated hemoglobin, ALP, systolic blood pressure, and length of time with diabetes than those in the non-DR group (*p* < 0.05).

**Table 1:** Demographic and sociological characteristics of participants grouped with or without diabetic retinopathy.

Variable	Without DR(n=3163)	With DR(n=840)	*P*-value
Age(years)	59.45 ± 13.35	59.28 ± 13.09	0.7535
Gender(%)			0.9188
Male	51.54	51.33	
Female	48.46	48.67	
Race(%)			0.1215
Mexican American	9.30	8.21	
Other Hispanic	5.38	5.46	
Non-Hispanic White	63.29	59.91	
Non-Hispanic Black	13.97	16.40	
Other Race - Including Multi-Racial	8.06	10.01	
Education(%)			0.0026
Less Than 9th Grade	9.57	12.75	
9-11th Grade (Includes 12th grade with no diploma)	12.79	14.74	
High School Grad/GED or Equivalent	24.62	26.69	
Some College or AA degree	32.58	30.41	
College Graduate or above	20.36	15.41	
Don’t know	0.08	0	
Smoke at least 100 cigarettes			0.7867
Y	51.08	49.77	
N	48.92	50.23	
Smoke now			0.8892
Every day	27.69	26.62	
Some days	3.31	3.12	
Not at all	69.00	70.26	
at least 12 alcohol drinks/1 yr			0.0052
Y	67.67	60.93	
N	32.20	39.07	
Don’t know	0.13		
Poverty income ratio (PIR)	2.81 ± 1.60	2.54 ± 1.63	<0.0001

DR, diabetic retinopathy.

**Table 2:** Biological characteristics of participants grouped with or without diabetic retinopathy.

Variable	Without DR(n=3163)	With DR(n=840)	*P*-value
BMI(kg/m^2)	33.11 ± 7.46	33.29 ± 8.04	0.5554
Waistline(cm)	111.47 ± 16.02	111.41 ± 17.20	0.9251
SBP (mmHg)	129.26 ± 18.60	131.60 ± 21.66	0.0030
DBP (mmHg)	69.38 ± 13.56	68.44 ± 15.01	0.0979
Duration of diabetes(years)	4.26 ± 71.01	13.39 ± 51.25	0.0008
HbA1c (%)	7.22 ± 1.66	7.71 ± 1.79	<0.0001
HDL (mmol/L)	1.22 ± 0.36	1.25 ± 0.36	0.0562
LDL(mmol/L)	2.57 ± 0.91	2.58 ± 1.02	0.8387
TG(mmol/L)	1.89 ± 1.97	1.88 ± 2.00	0.9400
TC(mmol/L)	4.68 ± 1.19	4.68 ± 1.26	0.9129
AST(U/L)	26.38 ± 21.57	24.54 ± 11.89	0.0234
ALT(U/L)	26.91 ± 32.29	24.24 ± 14.29	0.0258
ALP(U/L)	74.40 ± 31.51	77.89 ± 26.19	0.0045
γ-GGT(U/L)	35.18 ± 43.14	33.27 ± 35.45	0.2539
BUN(mg/dL)	16.05 ± 7.14	18.97 ± 10.87	<0.0001
Serum albumin(g/L)	41.37 ± 3.40	40.46 ± 3.58	<0.0001
Uric acid(umol/L)	339.15 ± 91.32	341.50 ± 104.11	0.5321
Serum creatinine(mg/Dl)	0.97 ± 0.57	1.18 ± 1.06	<0.0001

SBP, DBP: obtain three consecutive blood pressure readings after a 5-minute quiet rest in a seated position and after determining the participant’s maximum inflation level (MIL). We averaged the three BP readings to determine the SBP and DBP; HbA1c, glycated hemoglobin; HDL, high-density lipoprotein; LDL, low-density lipoprotein; TG, triglyceride; TC, total cholesterol; AST, aspartic transaminase; ALT, glutamic-pyruvic transaminase; ALP, alkaline phosphatase; γ-GGT, γ-glutamyltransferase; BUN, Blood urea nitrogen.

### Association of blood urea nitrogen levels with diabetic retinopathy

**Figure 2:**
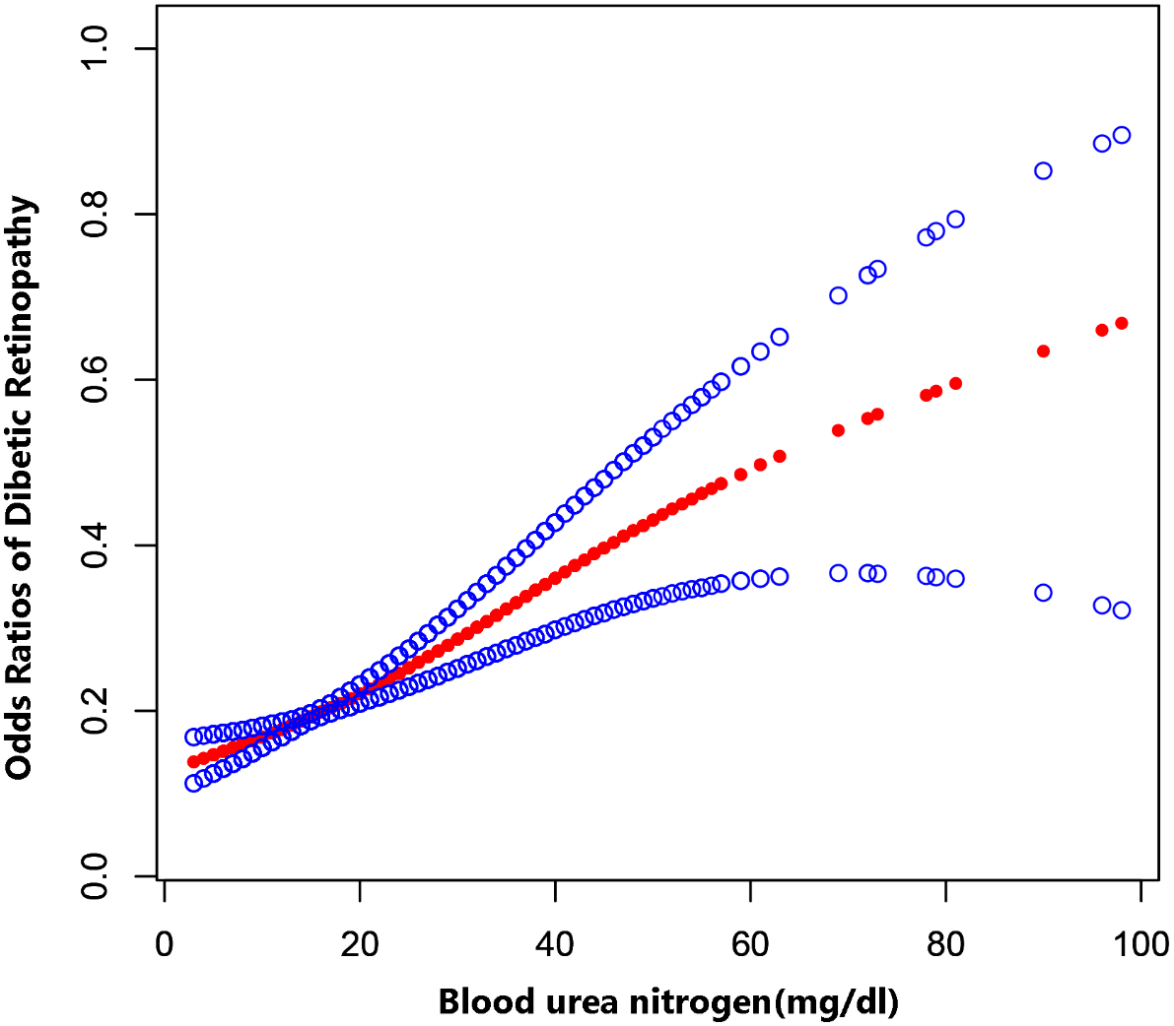
The association between BUN levels and DR. The solid red line represents the smooth curve fit between variables. Blue bands represent the 95% confidence interval from the fit.

The multiple logistic regression analysis (**Table 3**) revealed a positive correlation between blood urea nitrogen levels and DR in the unadjusted model [OR = 1.04, 95% CI: (1.03, 1.05), *p* < 0.0001]. Model 2 showed no change in correlation after controlling for age, sex, and race. After adjustment for all covariates, the two remained positively correlated [OR = 1.05, 95% CI: (1.03, 1.06), *p* < 0.0001). For further analysis, BUN levels were divide into four parts according to the number of patients enrolled so that the number of patients in each group remained close, with the lowest quartile serving as the control and the highest quartile serving as the comparison group. In model 1, Q4 was linked with a 2.2-fold increase in the incidence of DR compared with Q1 [OR = 2.20, 95% CI: (1.77, 2.73), *p* < 0.0001]. After correcting for covariates, the results of Models 2 and 3 produced comparable to Model 1, indicating a strong positive relevance between BUN levels and DR incidence. **Figure 2** demonstrated a favorable non-linear correlation between BUN levels and DR.

**Table 3:** Relationship with BUN and DR.

	Model 1*			Model 2*			Model 3*		
	OR	95% CI	*P* value	OR	95% CI	P value	OR	95% CI	*P* value
BUN(mg/dl)	1.04	(1.03,1.05)	<0.0001	1.04	(1.03,1.05)	<0.0001	1.05	(1.03,1.06)	<0.0001
Q1(3-11)	1(reference)			1(reference)			1(reference)		
Q2(12-14)	1.00	(0.78,1.28)	0.9897	0.97	(0.75,1.25)	0.8155	0.88	(0.66,1.19)	0.4105
Q3(15-19)	1.43	(1.14,1.78)	0.0020	1.36	(1.08,1.72)	0.0088	1.50	(1.15,1.96)	0.0029
Q4(20-98)	2.20	(1.77,2.73)	<0.0001	2.05	(1.62,2.60)	<0.0001	2.17	(1.65,2.87)	<0.0001
*P* for trend	1.06	(1.04,1.07)	<0.0001	1.05	(1.04,1.07)	<0.0001	1.06	(1.04,1.08)	<0.0001

*Model 1 does not contain any covariates. Model 2 contains only age, gender, and race. Model 3 includes all covariates(age, gender, race, poverty index, educational level, systolic blood pressure, diastolic blood pressure, BMI, waist circumference, glycosylated hemoglobin, serum albumin levels, triglycerides, total cholesterol, low-density lipoprotein (LDL) cholesterol, and high-density lipoprotein (HDL) cholesterol).

### Subgroup analyses

**Figure 3:**
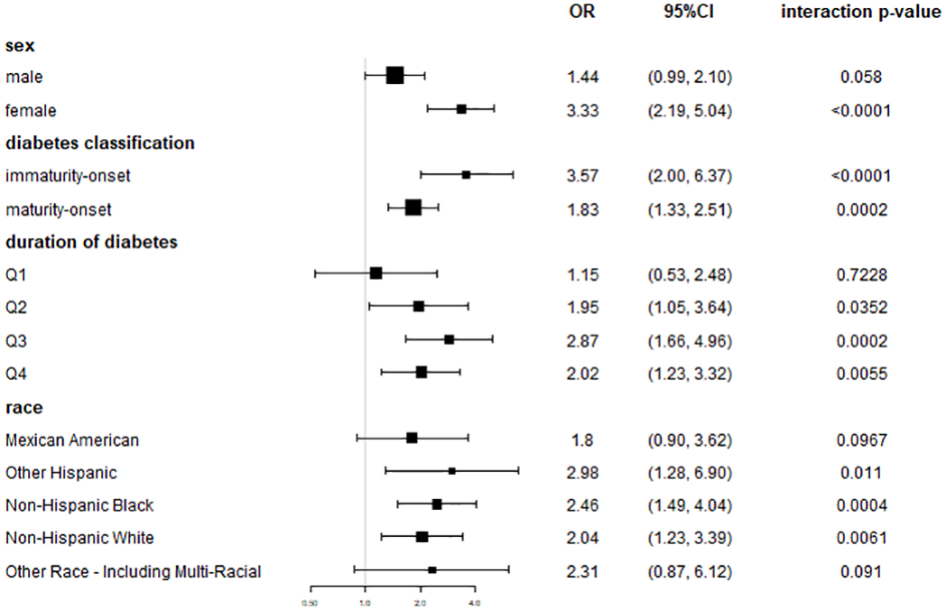
Coefficient plots of risk association between BUN and DR in subgroup of gender, diabetes classification, duration of diabetes and race. In model III, which adjusted for all covariates, the OR and 95% CI at the highest quartile of BUN (≥20 mg/dL), using the lowest quartile of BUN as a reference.

Subgroup studies stratified by sex revealed that blood urea nitrogen levels were positively correlated with DR occurrence in both men and women, with the link being larger in women. In model 3 (males), the incidence of DR was 1.44 times higher at Q4 levels of BUN than at Q1 [OR = 1.44, 95% CI: (0.99, 2.10), *p* = 0.0580]. In model 3 (females), the incidence of DR was 3.33 times higher at Q4 levels of BUN than at Q1 [OR = 3.33, 95% CI: (2.19, 5.04), *p*<0.0001] (**Figure 3**).

Diabetes was classified as immaturity onset or maturity onset based on whether the patient was under the age of 40 when diagnosed. Subgroup analyses showed a greater connection between BUN levels and the development of DR in individuals with immaturity -onset diabetes. In model 3 (immaturity -onset diabetes), the incidence of DR was 3.57 times higher at Q4 levels of BUN than at Q1 [OR = 3.57, 95% CI: (2.00,6.37), *p*<0.0001]. In model 3 (maturity onset diabetes), the incidence of DR was 1.83 times higher at Q4 levels of BUN than at Q1 [OR = 1.83, 95% CI: (1.33,2.51), *p* = 0.0002] (**Figure 3**).

Subgroup studies dividing the length of time with diabetes into four equal groups showed that the connection between BUN and the incidence of DR grew increasingly significant as the duration of time with diabetes increased. In model 3 (duration of diabetes Q1), the association wasn’t statistically significant [OR = 1.15, 95% CI: (0.53, 2.48), *p*=0.5915]. In model 3 (duration of time with diabetes Q3), the incidence of DR was 2.87 times greater at BUN levels in Q4 compared with Q1 [OR = 2.87, 95% CI: (1.66,4.96), *p*=0.0002]. In model 3 (Q4 diabetes duration), the incidence of DR was 2.04 times greater at Q4 levels of BUN compared with Q1 [OR = 2.04, 95% CI: (1.55,1.82), *p*<0.0001] (**Figure 3**).

In subgroup analyses stratified by race, the correlation between BUN and DR incidence varied slightly by race (**Figure 3**).

## Discussion

This research used population-based cross-sectional information from the NHANES database from 2005 to 2018. The results showed that after adjusting for all covariates, blood urea nitrogen was positively associated with the likelihood of DR. The relationship was affected by gender, type of diabetes (immaturity or maturity onset), ethnicity and duration of diabetes.

Several earlier studies have shown a correlation between BUN levels and DR. Mingzhi Zhang et al (19) did a multivariate logistic regression analysis of risk factor screening for diabetic retinopathy in China and discovered an association between BUN (OR, 1.012), serum creatinine (OR, 1.003) and DR. A study by Yingzi Li et al (20) found that diabetic patients with BUN greater than 8.2 mmol/L had reduced superficial retinal capillary density compared to those with BUN less than 8.2 mmol/L (*P* < 0.05). The clinical prediction model for diabetic nephropathy and diabetic retinopathy in diabetic patients developed by Fanhu et al (21) also included blood urea nitrogen as one of the risk factors. These findings are congruent with ours. Furthermore, a recent study used multivariate logistic analysis to confirm that BUN was highly correlated positively with the risk of DR and that the correlation was affected by the duration of diabetes (18). These studies included relatively insufficient sample size or a single source, whereas our examined cross-sectional data from multiple cycles of NHANES and adjusted the weights to make the conclusions more trustworthy. We conducted various subgroup analyses to further explore the relationship, which revealed that the association was related to gender, race, and age at diagnosis of diabetes, as well as being affected by diabetes duration. Subgroup analyses reveal a disparity in diabetic retinopathy (DR) risk between genders, with women exhibiting a heightened susceptibility compared to men, even when baseline blood urea nitrogen (BUN) levels are equated. Numerous investigations corroborate this gender discrepancy, delineating a greater prevalence and accelerated progression of DR in females (22, 23), particularly among those aged over 60 with longstanding diabetes (23). Experimental evidence further intimates that progesterone may instigate vascular endothelial growth factor upregulation (24), thereby fostering neoangiogenesis, a pivotal process in DR advancement. Conversely, supplemental estrogen administration to menopausal women appears to mitigate retinal neurovascular disease risk (25, 26), albeit the precise mechanistic underpinnings remain elusive (27). Notably, conflicting reports also cite male predisposition to DR, engendering ambiguity in gender-based conclusions (28). Concerning racial disparities, Hispanics manifest the highest DR susceptibility, trailed by blacks, findings congruent with extant literature (29, 30). Moreover, subgroup analyses underscore a pronounced DR risk escalation in individuals with an early diabetes onset (<=40 years). Multiple studies corroborate a correlation between younger diabetes onset and augmented diabetic complications (31, 32), positing elevated glycated hemoglobin levels and hastened pancreatic β-cell failure (33) as putative mechanisms, engendering formidable glycemic control challenges in this demographic.

Blood urea nitrogen is extensively utilized in the assessment and prognosis of renal insufficiency and heart failure, therefore many previous studies have concentrated on the connection between BUN and diabetic nephropathy (34) or diabetic adverse cardiovascular events (35). In recent years, studies on the correlation between BUN and DR have shown a positive correlation, implying that DR, diabetic nephropathy, and severe cardiovascular events may all have a shared etiology linked to elevated blood urea nitrogen levels. Previous studies have demonstrated the interdependence of DR with glycol-renal and cardiovascular disease and the existence,indicating a similar pathophysiology (36, 37). Oxidative stress is considered to be one of the primary pathogenic processes of vascular complications in diabetes (38), and DR, diabetic nephropathy (DN) and CVD are all prevalent microvascular and macrovascular complications of diabetes. Metabolic irregularities induced by hyperglycemia promote oxidative stress, which can stimulate stronger inflammation (39). Endothelial dysfunction is considered to be a shared pathogenic mechanism of DR and DN (40), and the glomerular endothelial barrier or blood-retinal endothelial barrier plays a key part in the pathogenic mechanism of DN and DR, which may also be the primary cause of elevated BUN levels in DR patients. In recent years, the study of gut flora has increasingly deepened, bring the association between DR, DN and diabetic cardiovascular disease even closer (41), and the gut flora has an impact on metabolism, intestinal barriers, and immune function (42), which may also contribute to the elevated BUN levels.

The novelty of our work is the inclusion of large-scale cross-sectional data from multiple time-period populations, as well as an in-depth exploration of the combined effects of gender, race, diabetes subtype, and diabetes duration on the BUN-DR relationship. At the same time, our study does have certain limitations. First, the diagnosis of diabetes and DR was on account of the results of a questionnaire survey, which led to some uncertainty in the final inclusion of individuals who may have been eligible. Second, because our study cohort was entirely from the United States, the findings should be interpreted with caution in other regions with significant disparities in living conditions or ethnicity.

In conclusion, our investigation revealed a favorable correlation between BUN and DR. As a result, BUN can be used to evaluate the risk of DR in diabetic patients, with the evaluating effect being particularly strong in women, patients with immaturity-onset diabetes mellitus, and patients with a lengthy history of the condition.

## Data availability statement

The original contributions presented in the study are included in the article/supplementary material. Further inquiries can be directed to the corresponding author.

## Author contributions

KD: Conceptualization, Data curation, Investigation, Software, Visualization, Writing – original draft, Writing – review & editing. WL: Funding acquisition, Project administration, Resources, Supervision, Validation, Writing – review & editing, Formal analysis, Methodology.
